# Response: Commentary: Effects of Age and Initial Risk Perception on Balloon Analog Risk Task: The Mediating Role of Processing Speed and Need for Cognitive Closure

**DOI:** 10.3389/fpsyg.2017.00541

**Published:** 2017-04-11

**Authors:** Szymon Wichary, Thorsten Pachur, Maciej Kościelniak, Klara Rydzewska, Grzegorz Sedek

**Affiliations:** ^1^Wrocław Faculty of Psychology, SWPS University of Social Sciences and HumanitiesWroclaw, Poland; ^2^Max Planck Institute for Human DevelopmentBerlin, Germany; ^3^Department of Psychology, Interdisciplinary Center for Applied Cognitive Studies, SWPS University of Social Sciences and HumanitiesWarsaw, Poland

**Keywords:** aging, risk-taking, balloon analog risk task (BART), cognitive modeling, Bayesian analysis

In a study on aging and risk taking with a large female sample, Koscielniak et al. (2016) found age differences in the Balloon Analogue Risk Task (BART; Lejuez et al., 2002), a popular measure of risk taking. In this task, older participants were more risk averse than younger participants, as indicated by a lower number of balloon pumps and balloon explosions. Furthermore, an experimental manipulation of the time point of balloon explosion in the first three trials of the task (specifically, there was a bad and a good luck condition) influenced behavior in subsequent trials: after having experienced initial trials with early explosions participants were more risk averse than after having experienced initial trials with late explosions. The authors interpreted these results from a dual-process theoretical perspective (e.g., Hess, 2015).

In a commentary on this article, Walasek (2016) highlighted the need of using computational modeling instead of verbal frameworks in order to understand the mechanism underlying the impact of age on decision making. Here, we respond to Walasek's commentary by providing a computational modeling analysis of Koscielniak et al.'s (2016) data. Specifically, we applied the Bayesian Sequential Risk (BSR) model, which allows to decompose behavior in the BART task (Wallsten et al., 2005; Pleskac, 2008; see also Wichary et al., 2015) into four psychological components (represented by model parameters): reward sensitivity (γ^+^), choice consistency (β), the initial belief that the balloon will not explode (${\hat{q}}_{I}$), and the uncertainty in that initial belief (δ), which can also be interpreted as a learning rate. We implemented the model using a hierarchical Bayesian approach, which yields posterior distributions of the model parameters (see Supplementary Materials for details). The decomposition of people's behavior on the BART therefore allows one to gain insights into which psychological processes are affected specifically by the experimental manipulation and which differ between the age groups—and thus drive the differences observed on the behavioral level. For instance, is the decreased risk taking observed in the bad luck condition due to a motivational factor (decreased reward sensitivity) or a cognitive factor (belief about the explosion probability), or both?

Figure 1 shows the posterior distributions of the group-level means of the four BSR parameters, separately for the younger and older individuals and for the good luck vs. bad luck conditions. As can be seen, the manipulation of whether in the initial trials of the BART participants experienced very early or rather late explosions had both an impact on the parameter ${\hat{q}}_{I}$, capturing the initial belief that the balloon will not explode, and the reward sensitivity (parameter γ^+^), though more pronounced for the former than for the latter. Specifically, the ${\hat{q}}_{I}$ parameter was higher in the good luck than in the bad luck condition, and this held both for the younger and the older adults; additionally, the γ^+^ parameter was lower in the good luck than in the bad luck condition; this was the case in particular for the younger adults, whereas for the older adults the highest density interval (expressing the uncertainty in the estimates) of the differences included zero (see Supplementary Material for details). Further, δ (here log-transformed for better readability, with higher values indicating higher uncertainty) was higher in the bad luck than in the good luck condition, indicating greater uncertainty in the initial belief that the balloon will not explode and thus also more pronounced learning in the former. One likely reason for this latter difference is that the discrepancy between the explosion probability that participants experienced in the initial (i.e., manipulated) and in subsequent trials was somewhat larger in the bad luck than in the good luck condition. As regards age differences, the model parameters indicated differences in the reward sensitivity parameter, which was lower for the older than the younger adults in the good luck condition (but not in the bad luck condition). None of the other parameters showed credible differences between the age groups; nevertheless, it should be noted that in line with existing analyses (e.g., Pachur et al., in press), in both the good and bad luck conditions there was a pattern of older adults showing lower choice consistency (parameter β) than younger adults, but based on the current data these differences were not credible.

**Figure 1:**
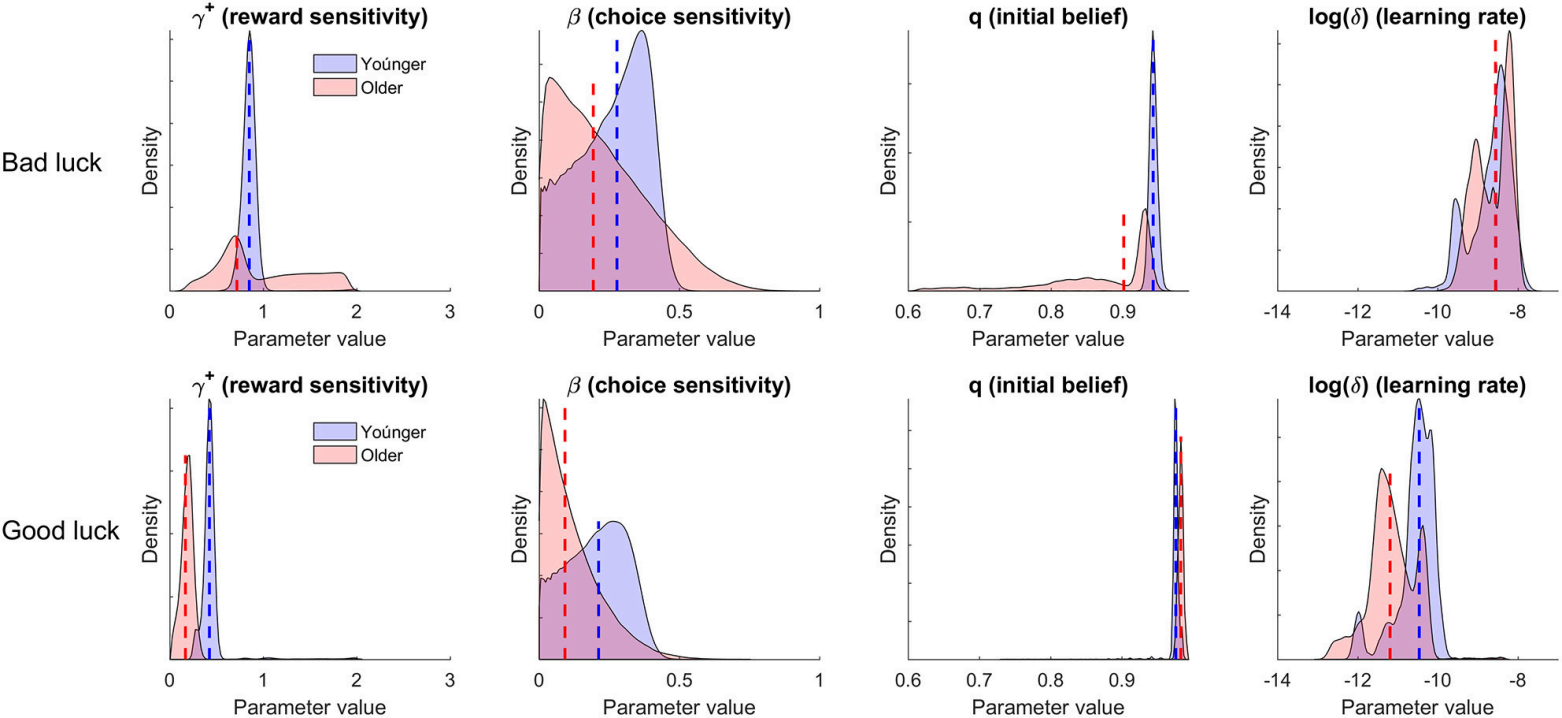
**Results from the hierarchical Bayesian analysis of the data of Koscielniak et al. (2016) with the Bayesian Sequential Risk (BSR) model**. Shown are the posterior group-level distributions of the parameters, separately for the younger and older participants and for the bad luck and good luck conditions. The broken lines indicate the median.

## Discussion and conclusions

In a response to the commentary by Walasek (2016), we conducted a computational modeling analysis of Koscielniak et al.'s (2016) data, which provides a complementary explanation to the verbal, dual-process account used to interpret the results in that study. Our analysis allowed to disentangle the cognitive and motivational mechanisms underlying the impact of aging and a manipulation of the initial learning experiences on risk taking, as measured with the BART. The analysis revealed how age and the experimental manipulation influenced these parameters and the psychological parameters they represent. For instance, the age differences in BART performance were paralleled by differences in the reward sensitivity parameter, indicating an importance of this mechanism for understanding the impact of age on risk taking. Moreover, different initial experiences in the task impacted people's initial beliefs about the balloon, but also the uncertainty (or learning rate) during the subsequent learning process. Finally, reward sensitivity was higher when participants initially experienced early balloon explosions. This difference might seem puzzling, as it appears at odds with the common association between higher reward sensitivity and *higher* (not lower, as observed in the bad luck condition) risk seeking. From a psychophysical perspective, however, this result makes sense. People's initial experiences in the bad luck condition (in which they ended up with, if at all, very small rewards) might have anchored them on small rewards, such that they were more sensitive to the subsequent higher rewards than in the good luck condition.

In sum, the conclusions we can draw from this computational modeling of risk behavior are more specific with regard to the underlying cognitive and motivational processes than in the original analyses, that focused solely on behavior. Such detailed conclusions can fuel precise theorizing about the mechanism linking aging and risk taking, which, eventually, might supplement verbal theoretical frameworks, as suggested by Walasek's (2016) commentary.

## Author contributions

Substantial contributions to the statistical analysis and interpretation of data (TP, SW). Substantial contributions to the design of the work and data acquisition (MK, KR, GS). Drafting the work (SW, TP, MK, KR, GS). Final approval of the version to be published (SW, TP, MK, KR, GS). Agreement to be accountable for all aspects of the work (SW, TP, MK, KR, GS).

## Funding

This work was supported by the National Science Centre, Poland, under Grant 2015/17/B/HS6/04185, awarded to GS.

### Conflict of interest statement

The authors declare that the research was conducted in the absence of any commercial or financial relationships that could be construed as a potential conflict of interest.
