# Supplementary material for: Response: Commentary: Effects of Age and Initial Risk Perception on Balloon Analog Risk Task: The Mediating Role of Processing Speed and Need for Cognitive Closure
Source: Front Psychol. 2017 Apr 11;8:541. doi: 10.3389/fpsyg.2017.00541 (PMC5387090; doi:10.3389/fpsyg.2017.00541)
Supplement: Supplementary file 1 [file DataSheet1.doc]

Supplementary Material

*Response: "Commentary: Effects of Age and Initial Risk Perception on Balloon Analog Risk Task: The Mediating Role of Processing Speed and Need for Cognitive Closure*”

Szymon Wichary^1^, Thorsten Pachur^2^, Maciej Kościelniak^3^, Klara Rydzewska^3^, Grzegorz Sędek^3*^

^1^SWPS University of Social Sciences and Humanities, II Faculty of Psychology, Wroclaw, Poland

^2^Max Planck Institute for Human Development, Berlin, Germany

^3^SWPS University of Social Sciences and Humanities, Interdisciplinary Center for Applied Cognitive Studies, Warsaw, Poland

*** Correspondence:** Grzegorz Sedek

SWPS University

Chodakowska 19/31

00-183 Warsaw, Poland

gsedek@swps.edu.pl

**Formal Description of the Bayesian Sequential Risk-Taking (BSR) Model**

The BSR assumes that people’s behavior in a sequential risk-taking task such as the BART is primarily a function of three cognitive processes: learning, evaluation, and response selection. With regard to learning processes, the BSR assumes that decision makers have an initial belief in the probability $\hat{q}_{h}$ that balloon *h* will not explode on any given pump. This belief is modeled with a beta distribution over $\hat{q}_{h}$, summarized by two parameters *a_h_* > 0 and *b_h_* > 0, which are estimated from the data. A quantification of decision makers’ beliefs in the chances of balloon *h* not exploding is derived from the mean of the beta distribution, defined as follows (see Pleskac, 2008):

$\hat{q}=\frac{a_{h}}{a_{h}+b_{h}}$ (1)

The variance of the distribution, which can be interpreted as indicating the decision makers’ uncertainty in the initial belief, is determined as

$\delta_{h}=\frac{a_{h}b_{h}}{(a_{h}+b_{h})^{2}(a_{h}+b_{h}+1)}$. (2)

Higher values of *δ_h_* translate into a greater adjustment after experiencing the trial outcome (i.e., whether the balloon bursts or not); the parameter can therefore also be viewed as a learning rate, with higher values indicating stronger learning. For readability, *δ*_h_ is sometimes log-transformed (e.g., Pleskac, 2008). The decision maker’s initial belief that the balloon will burst as well as the initial uncertainty in the belief, $\hat{q}_{1}$ and δ_1_, are estimated from the data by estimating *a*_1_ and *b*_1_.

The second key aspect of the BSR refers to the decision maker’s reward sensitivity, that is, how the outcome experienced after each pump opportunity (i.e., whether the balloon bursts or not) impacts the evaluation of pumping at the subsequent pump opportunity. Specifically, the expected gain of pumping balloon *h* at opportunity *i* equals

$v_{hi}=(\hat{q}_{h})^{i}(ix)^{\gamma^{+}}$ (3)

The variable $\hat{q}_{h}$is the probability that balloon *h* will not explode after *i* pumps, and *x* is the reward for each successful pump. Higher values of γ^+^ indicate greater sensitivity to differences in payoffs. Participants are assumed to have a target of *c* pumps, which maximizes the expected payoffs. This target number of pumps is defined as the maximum of Equation 3 (see Wallsten et al., 2005), which is

. (4)

Finally, the BSR models how reward evaluation and learning experience are translated into the probability *r*_i_ that the balloon is pumped another time:

$r_{i}=\frac{1}{1+e^{\beta d_{hi}}}$, (5)

where β is a free parameter representing how consistently participants follow their target number of pumps (i.e., *G*_h_). Lower values of β indicate that the decision maker’s pump tendency is sensitive to other information besides his or her targeted reward pump (or is just noisy) and that the pumping behavior will thus be more variable. *d*_h_(*i*) is the distance at opportunity *i* from the targeted number of pumps, *d*_h_(i) = *i* – *G*_h_.

**Details of the Bayesian Parameter Estimation Procedure**

The four parameters of the BSR model, γ^+^, β, *a_h_*, and *b_h_*, were estimated using a hierarchical Bayesian approach, separately for the age groups and experimental condition. In Bayesian parameter estimation, estimates of the parameter values are initially represented in terms of *prior* distributions, which are then updated into *posterior* distributions in light of the data (for an introduction, see Lee & Wagenmakers, 2013). The posterior distributions represent the uncertainty in the parameter estimate and can be summarized in statistics such as the mean and the 95% highest density interval. The advantage of a hierarchical approach to parameter estimation is that individual parameters are partially pooled through group-level distributions, thus yielding more reliable estimates than the traditional, nonhierarchical approach (e.g., Kruschke & Vanpaemel, 2015).

The individual-level parameters were assumed to be drawn from normally distributed group-level distributions, each represented with a mean and a standard deviation. The priors the means were set to uniform probability distributions spanning a reasonable range. Specifically, the ranges were 0 to 2 for γ^+^, 0 to 10 for β, 0 to 20,000 for *a_h_* and 0 to 500 for *b_h_*. The priors on the group-level standard deviations were uniformly distributed, ranging from 0 to 10 (thus avoiding extreme bimodal distributions on the individual level).

The joint posterior parameter distributions were estimated using Monte Carlo Markov Chain methods implemented in JAGS, a sampler that utilizes a version of the BUGS programming language (version 3.3.0) called from Matlab. We recorded a total of 24,000 samples, distributed across 12 chains, which were drawn from the posterior distributions after a burn-in period of 1,000 samples. To reduce autocorrelations during the sampling process, we recorded only every 100^th^ sample. The sampling procedures were efficient, as indicated by low autocorrelations of the sample chains, Gelman–Rubin statistics, and visual inspections of the chain plots.

We compared the means and distributions of the four BSR parameters (with the *a_h_*, and *b_h_*, parameters used to compute $\hat{q}_{1}$ and δ_1_ according to Equations 1 and 2) separately for the younger and older individuals and for the two conditions (good luck vs. bad luck). Table S1 presents the estimated means of the four parameters with the 95% highest confidence intervals for the both age groups and two experimental conditions. Table S2 presents the differences of the parameters between the age groups and conditions, respectively, along with the 95% highest confidence intervals for the both age groups and two experimental conditions.

Table S1

*Description of the parameters of the BSR model, their estimated means, and 95% highest confidence interval (in brackets), separately for younger and older adults and for the bad luck and good luck conditions*

| Parameter | Description | Bad luck | | Good luck | |
| --- | --- | --- | --- | --- | --- |
|  |  | Younger adults | Older adults | Younger adults | Older adults |
| γ^+^ | Measure of reward sensitivity. Higher values lead to a higher number of targeted pumps and thus more pumps being made on average. | 0.855 [0.701, 0.985] | 0.933 [0.111, 1.944] | 0.442 [0.275, 0.807] | 0.172 [0.026, 0.289] |
| β | Measure of how consistently participants follow their targeted evaluation. Lower values of β indicate that the participant’s decision to pump is sensitive to other information beside their pump target and is thus more variable. | 0.258 [0.019, 0.44] | 0.225 [0.008, 0.599] | 0.204 [0.014, 0.38] | 0.119 [0.004, 0.377] |
| $\hat{q}_{I}$ | Index of initial belief that the balloon will not explode. Higher values of $\hat{q}_{I}$ lead to a higher number of targeted pumps and thus more pumps being made on average. | 0.941 [0.931, 0.954] | 0.866 [0.648, 0.941] | 0.975 [0.955, 0.985] | 0.982 [0.976, 0.99] |
| log(δ) | Measure of uncertainty participants have in their initial belief about the likelihood of the balloon exploding. Higher values indicate more uncertainty and thus more sensitivity to observed pump data. | -8.68 [-9.705, -7.94] | -8.626 [-9.455, -8] | -10.55 [-11.995, -9.908] | -11.146 [-12.435, -10.224] |
| DIC | Model fit | 7,605.12 | 6,816.57 | 8,922.91 | 7,641.77 |

Note. DIC = deviance information criterion

Table S2

*Difference on the BSR model parameters the between the age groups and conditions, respectively, 95% highest confidence interval (in brackets) of the differences. The differences are reported separately for the bad luck and good luck conditions, and younger and older adults, respectively*

| Parameter | Younger adults vs. older adults | | | Bad luck vs. good luck | |
| --- | --- | --- | --- | --- | --- |
|  | Bad luck | Good luck | Younger adults | | Older adults |
| γ^+^ | -0.078 [-1.105, 0.75] | 0.27 [0.091, 0.571] | 0.413 [0.106, 0.627] | | 0.761 [-0.064, 1.779] |
| β | 0.033 [-0.411, 0.365] | 0.085 [-0.233, 0.328] | 0.053 [-0.268, 0.349] | | 0.105 [-0.251, 0.515] |
| $\hat{q}_{I}$ | 0.076 [-0.002, 0.294] | -0.007 [-0.029, 0.004] | -0.033 [-0.048, -0.015] | | -0.116 [-0.334, -0.041] |
| log(δ) | -0.054 [-1.494, 1.184] | 0.596 [-0.9, 1.534] | 1.87 [0.478, 3.679] | | 2.52 [1.018, 4.2] |

**References**

Pleskac, T. J. (2008). Decision making and learning while taking sequential risks. *Journal of Experimental Psychology: Learning, Memory, and Cognition*, *34*, 167-185.

Kruschke, J. K., & Vanpaemel, W. (2015). Bayesian estimation in hierarchical models. In J. R. Busemeyer, Z. Wang, J. T. Townsend, & A. Eidels (Eds.), *The Oxford handbook of computational and mathematical psychology* (pp. 279‒299). Oxford, United Kingdom: Oxford University Press.

Lee, M. D., & Wagenmakers, E.-J. (2013). *Bayesian cognitive modeling: A practical course.* Cambridge, United Kingdom: Cambridge University Press.

Wallsten, T. S., Pleskac, T. J., & Lejuez, C. W. (2005). Modeling behavior in a clinically diagnostic sequential risk-taking task. *Psychological Review*, *112*, 862 -880 .
